# Genetic loci of beta-aminoisobutyric acid are associated with aging-related mild cognitive impairment

**DOI:** 10.1038/s41398-023-02437-y

**Published:** 2023-04-29

**Authors:** Einat Granot-Hershkovitz, Brian Spitzer, Yunju Yang, Wassim Tarraf, Bing Yu, Eric Boerwinkle, Myriam Fornage, Thomas H. Mosley, Charles DeCarli, Bruce S. Kristal, Hector M. González, Tamar Sofer

**Affiliations:** 1 Division of Sleep and Circadian Disorders, Brigham and Women’s Hospital, Boston, MA USA; 2 Department of Medicine, Harvard Medical School, Boston, MA USA; 3 Brown Foundation Institute of Molecular Medicine, McGovern Medical School, The University of Texas Health Science Center at Houston, Houston, TX USA; 4 Institute of Gerontology, Wayne State University, Detroit, MI USA; 5 Human Genetics Center, School of Public Health University of Texas Health Science Center at Houston, Houston, TX USA; 6 Human Genome Sequencing Center, Baylor College of Medicine, Houston, TX USA; 7 Department of Neurology, School of Medicine, University of Mississippi Medical Center, Jackson, MS USA; 8 Alzheimerʼs Disease Center, Department of Neurology, University of California, Davis, Sacramento, CA USA; 9 Department of Neurosciences, University of California, San Diego, La Jolla, CA USA; 10 Department of Biostatistics, Harvard T.H Chan School of Public Health, Boston, MA USA; 11 Division of Cardiology, Beth Israel Deaconess Medical Center, Boston, MA, USA

**Keywords:** Predictive markers, Genomics, Diseases

## Abstract

We studied the genetic associations of a previously developed Metabolomic Risk Score (MRS) for Mild Cognitive Impairment (MCI) and beta-aminoisobutyric acid metabolite (BAIBA)—the metabolite highlighted by results from a genome-wide association study (GWAS) of the MCI-MRS, and assessed their association with MCI in datasets of diverse race/ethnicities. We first performed a GWAS for the MCI-MRS and BAIBA, in Hispanic/Latino adults (*n* = 3890) from the Hispanic Community Health Study/Study of Latinos (HCHS/SOL). We identified ten independent genome-wide significant (*p* value <5 × 10^−8^) variants associated with MCI-MRS or BAIBA. Variants associated with the MCI-MRS are located in the Alanine-Glyoxylate Aminotransferase 2 (*AGXT2* gene), which is known to be associated with BAIBA metabolism. Variants associated with BAIBA are located in the *AGXT2* gene and in the *SLC6A13* gene. Next, we tested the variants’ association with MCI in independent datasets of *n* = 3178 HCHS/SOL older individuals, *n* = 3775 European Americans, and *n* = 1032 African Americans from the Atherosclerosis Risk In Communities (ARIC) study. Variants were considered associated with MCI if their *p* value <0.05 in the meta-analysis of the three datasets and their direction of association was consistent with expectation. Rs16899972 and rs37369 from the *AGXT2* region were associated with MCI. Mediation analysis supported the mediation effect of BAIBA between the two genetic variants and MCI (*p* value = 0.004 for causal mediated effect). In summary, genetic variants in the *AGXT2* region are associated with MCI in Hispanic/Latino, African, and European American populations in the USA, and their effect is likely mediated by changes in BAIBA levels.

## Introduction

Hispanic/Latino older adults suffer from a higher risk for mild cognitive impairment (MCI) compared to non-Hispanic White adults and are a rapidly growing ethnic population in the United States [1]. MCI is an early stage of decline in abilities across any cognitive domains such as memory, attention, language, executive function, visuospatial skill, or perceptual skill not affecting activities of daily living. MCI can result from genetic susceptibility and/or lifestyle, and environmental risk factors [2]. Pathophysiological changes underlining MCI may occur years before clinical symptoms appear, thus providing a potential window to detect and facilitate interventions at earlier stages of the disease [3]. However, very little work has been done on the discovery of genetic determinants of MCI. Rather, many previous studies focused on assessing genetic risk for Alzheimer’s Disease (AD) with MCI [4], or genetic risks associated with the conversion of MCI cases to AD [5, 6]. In the Study of Latinos—investigation of Neurocognitive Aging (SOL-INCA), we previously saw that MCI was not associated with the strongest AD genetic risk factor, the *APOE*-*∈*4 allele [7, 8], while an AD polygenic risk score constructed using single nucleotide polymorphisms (SNPs) mainly from the *APOE* region, was associated with MCI [9]. Thus, while AD genetic risk factors are sometimes associated with MCI, approaches leveraging other risk factors for cognitive aging are needed to facilitate genetic discoveries for MCI. This is even more important in Hispanic/Latino populations, where AD pathology is a less common cause of MCI compared to White populations.

In the last decade, metabolome assessment has emerged as a new approach for biomarker discovery, and for evaluating the progress of disease and its underlying pathophysiology [10]. Recent studies have demonstrated metabolic dysregulation in individuals with MCI or dementia [11], and prospective studies explored risk prediction for MCI based on metabolite biomarkers [12, 13]. We recently developed a metabolomic risk score (MRS) for Hispanic/Latino older adults in the United States, predicting MCI identified 7 years after metabolomics assessment [14] in individuals from SOL-INCA. Building on metabolomics and other omics associations with measured traits, emerging approaches use genetic determinants of such omics measures to identify causal pathways underlying phenotypes. For example, other researchers utilized SNP associations with many measured proteins to perform phenome-wide Mendelian randomization analysis to detect genetic determinants, mediated with changes in protein levels of various phenotypes [15], or utilized metabolite predictors of type 2 diabetes [16].

**Fig. 1:**
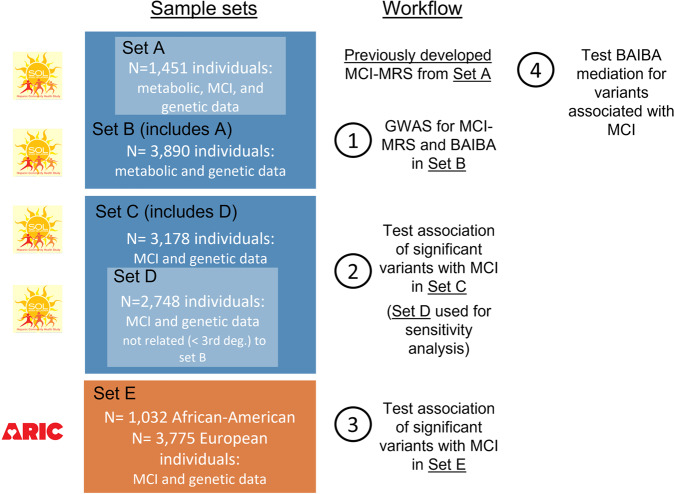
Analysis flowchart and the corresponding HCHS/SOL and ARIC analytic datasets. We performed a multi-step analysis. Step 1: Identification of genetic associations with the MCI-MRS and BAIBA. Step 2: Association testing of the significantly associated genetic variants with MCI in a separate subset of US Hispanic/Latino older adults. Step 3: Assessment of generalizability of the genetic associations with MCI in European and African Americans from the Atherosclerosis Risk in Communities (ARIC) study. Step 4: Assessment of the mediation effect of BAIBA in the association of the genetic variants with MCI.

We hypothesize that genetic determinants influencing the MCI-MRS may also influence MCI, either through a pleiotropic pathway or by a mediation pathway where MRS, or any of its metabolite components, is a mediator between the genetic determinants and MCI. The analysis steps are described in Fig. 1. We first identified genetic associations with the MCI-MRS and BAIBA (the MRS metabolite highlighted by the MCI-MRS GWAS results), which provide insights into the biological basis and heritability of the MCI-MRS (Step 1). Next, we tested the association of the significantly associated genetic variants with MCI in a separate subset of participants drawn from SOL-INCA (Step 2). We further assessed the generalizability of these genetic associations with MCI in European and African American participants from the Atherosclerosis Risk In Communities (ARIC) study (Step 3). Next, we assessed the mediation effect of BAIBA in the association of the genetic variants with MCI (Step 4), and further determined the association of lifestyle characteristics on MCI-MRS and BAIBA.

## Methods

### Study population

The HCHS/SOL is a population-based longitudinal multi-site cohort study of Hispanic/Latino adults in the United States. The study primarily enrolled participants from six self-identified Hispanic/Latino backgrounds: Cuban, Central American, Dominican, Mexican, Puerto Rican, and South American [17, 18]. A total of 16,415 adults, 18–74-year-old, were enrolled in the baseline visit at four field centers (Bronx, NY, Chicago, IL, Miami, FL, and San Diego, CA) (2008-2011). A detailed description of the sampling design, including the generation and use of survey weights for the HCHS/SOL, was previously published [17, 18]. Cognitive function was assessed in 9714 individuals aged 45 years or older during the baseline visit. The Study of Latinos-Investigation of Neurocognitive Aging (SOL-INCA) is an ancillary study of HCHS/SOL, focusing on the middle-aged and older adult group who underwent cognitive assessment at visit 1 [19]. Overall, 6377 individuals 50 or older with baseline cognitive testing participated in the SOL-INCA examination, taking place at or after HCHS/SOL visit 2, with an average of 7 years since visit 1. Metabolites were measured in serum, after fasting, on a random subset of 3978 HCHS/SOL participants from visit 1, and profiling was done using untargeted liquid chromatography-mass spectrometry (LC-MS) using the discovery HD4 platform in 2017 at Metabolon Inc. (Durham, NC).

All participants in this analysis signed written informed consent in their preferred language (Spanish/English). The HCHS/SOL was approved by the institutional review boards (IRBs) at each field center, where all participants gave written informed consent, and by the Non-Biomedical IRB at the University of North Carolina at Chapel Hill, to the HCHS/SOL Data Coordinating Center. All IRBs approving the study are Non-Biomedical IRB at the University of North Carolina at Chapel Hill. Chapel Hill, NC; Einstein IRB at the Albert Einstein College of Medicine of Yeshiva University. Bronx, NY; IRB at Office for the Protection of Research Subjects (OPRS), University of Illinois at Chicago. Chicago, IL; Human Subject Research Office, University of Miami. Miami, FL; Institutional Review Board of San Diego State University, San Diego, CA. The present study was approved as a secondary data analysis protocol by the Mass General Brigham IRB protocol #2019P000057.

### Neurocognitive outcomes

We studied prevalent MCI at the SOL-INCA visit, classified according to National Institute on Aging-Alzheimer’s Association criteria [20]. In brief, the SOL-INCA MCI research diagnostic operational definition [7, 19] included three criteria: [1] a cognitive test score below –1 standard deviation (SD) in any of the cognitive tests applied at the SOL-INCA exam, where means and SDs were based on SOL-INCA robust internal norms, [2] a rate of global cognitive decline between the HCHS/SOL baseline and the SOL-INCA exam of than −0.055 SD or more per year, and [3] any self-reported subjective cognitive decline using the Everday Cognition 12-item version (E-Cog12) [21]. Additionally, individuals were classified as MCI+ if they met two conditions: (a) a cognitive test performance below –2 SD in any SOL-INCA neurocognitive test, and (b) more than minimal impairment in the instrumental activities of daily living (IADL) [22].

### Metabolomic risk score (MRS) for MCI

We previously developed an MRS for MCI based on selected fasting serum metabolites, from a LASSO-penalized regression [23] using 1451 SOL-INCA individuals who also had metabolite measures [14]. The MRS forms a combined measure of the joint effect of 61 metabolites in predicting MCI. The MRS is defined as a weighted sum of metabolite values, of the form, for participant *i*:

$$mrs_i = \mathop {\sum }\limits_{j = 1}^{61} w_jm_{ij},$$

where *m*_*ij*_ is the level of the *j* metabolite in participant *i*, and *w*_*j*_ is the weight of the metabolite. The list of metabolites and weights is provided in Supplementary Table 1. Based on the metabolites and their weights, we constructed the MRS for 3968 HCHS/SOL individuals with metabolomics data. All metabolites used in the MRS had less than 25% missing values. They were treated as continuous and missing values were imputed using half of the lowest value observed in the sample per metabolite, under the assumption that missing values are due to metabolite concentration being below the limit of detection (i.e., missing not at random). Because some metabolites have skewed distribution, we originally rank-normalized the metabolites before summing them in the MRS, and scaled them back to their original scale by multiplying them by their standard deviations (SD), estimated prior to rank-normalization. We also adapted the weights according to the SDs estimated on the sample used for developing the MRS.

### Genotyping

*APOE* genotyping was performed using commercial TaqMan assays previously described [24]. For individuals with missing *APOE* genotypes, we computed *APOE* genotypes based on phased whole-genome sequencing (WGS) data from TOPMed Freeze 8. Other genetic data were used based on genotyping (rather than WGS) using an Illumina custom array, as previously reported [25]. Genome-wide imputation was conducted using the multi-ethnic NHLBI Trans-Omics for Precision Medicine (TOPMed) freeze 8 reference panel (GRCh38 assembly) [25]. Principal components (PCs) were previously computed using PC-Relate [26], and the kinship matrix was computed using the genetic data. “Genetic analysis groups” were constructed based on a combination of self-identified Hispanic/Latino backgrounds and genetic similarity, and are classified as Central American, Cuban, Dominican, Mexican, Puerto Rican, and South American [27].

### Heritability estimation for MCI-MRS and BAIBA

Heritability of the MRS and BAIBA (the MRS metabolite highlighted by the MCI-MRS GWAS results, see further details below) were estimated via a mixed model using the variance explained by the kinship matrix, representing the variance explained by additive effects of common genetic variants. Heritability was estimated in 3496 HCHS/SOL individuals (from Fig. 1, set B), after excluding >3rd-degree relatives estimated via the kinship coefficient.

### Genome-wide association studies (GWAS) for MCI-MRS and BAIBA

We performed MCI-MRS and BAIBA GWAS in 3890 HCHS/SOL individuals who had both genetic data and an MCI-MRS score and 3863 individuals with BAIBA values (27 individuals had missing BAIBA values) (Fig. 1, Step 1). We used the linear mixed model approach from the “GENESIS” R package and adjusted for age, sex, center, genetic analysis groups, first five PCs of genetic data, and random effects for kinship, household, and block unit. For both GWAS, we removed genetic variants with low minor allele count (MAC) (<60, corresponding to MAF ≲0.77%), and/or low imputation quality (*R*^2^ < 0.6), resulting in 12,518,657 variants in MCI-MRS GWAS and 12,481,432 in BAIBA GWAS. We used a two-stage method, in which we first regressed the trait on covariates, obtained residuals, rank-normalized them, and then used the rank-normalized residuals in the association with the genotypes [28], adjusting for the same covariates again. We applied a genome-wide significance threshold of *p* value = 5 × 10^−8^. Notably, due to applying the two-stage rank-normalization approach, the selected MAC threshold was expected to result in appropriate type 1 error control. Two-sided *p* values were computed using the score test.

When multiple variants within a genomic region (1 Mb window) were significantly associated with the MRS or BAIBA *(p* value <5 × 10^−8^), we conducted conditional analyses using the index (most significant) SNP as a covariate. If any of the remaining variants had associations with *p* value <5 × 10^−8^, we repeated this process, adding the top remaining variant to the model. We report the associations for independent SNPs based on the first discovery model. Finally, we assessed whether the findings from our BAIBA GWAS are similar to previously reported findings by looking up associations of SNPs from regions identified in other GWAS.

We computed the trait variance explained by the identified, genome-wide significant variants for each of the MCI-MRS and BAIBA, by comparing the total variance of a linear mixed model fitted to the metabolite outcome (MCI-MRS or BAIBA) with covariates age, sex, center, genetic analysis groups, first five PCs of genetic data, to the total variance of a similar model that also has the identified variants as covariates. The total variance was defined as the sum of the variance components corresponding to the kinship, household, and block unit matrices, and the residual variance of each model. The percent explained variance was defined as the percent reduction in total variance between the model with and without genetic variants.

### MCI-MRS-associated SNPs and their associations with MCI-MRS metabolites

While we focused on BAIBA because the single association region of the MCI-MRS encompasses the *AGXT2* gene known to be strongly associated with BAIBA, we also estimated genetic associations of the two MCI-MRS SNPs from the *AGXT2* region with all metabolites composing the MCI-MRS. We used the same linear mixed model approach as for the MCI-MRS and BAIBA GWAS, while focusing only on the two SNPs.

### Genetic association analysis with MCI in a separate HCHS/SOL dataset

We tested the association between the variants significantly associated with the MCI-MRS or BAIBA levels, and MCI in a set of 3149 HCHS/SOL individuals who were not included in the dataset used for the construction of the MCI-MRS (due to lack of metabolite data) (Fig. 1, Step 2). We employed the mixed model approach with a logistic link function and with the same covariates and random effects as described above. We stratified the analysis by the *APOE*-ε4 carrier status since the association of BAIBA and MCI was driven by the *APOE*-ε4 carrier stratum [14]. In a second model, we further included *APOE*-ε4 and *APOE*-ε2 carrier status as covariates. Associations were considered significant if they had a *p* value <0.05. *P* values were two-sided and were based on the score test. We note that family-wise error rate (FWER) control requires *p* value threshold accounting for all tested associations, i.e., 0.05/10 = 0.005. Finally, we performed a sensitivity analysis where we applied the same analysis on a smaller subset of 2748 individuals who are genetically unrelated to those who participated in the GWAS of the MCI-MRS and of BAIBA (individuals with >3rd-degree relatedness estimated via the kinship coefficient were excluded; Fig. 1, Step 3). This sensitivity analysis addresses the possibility that replicated genetic associations are potentially driven by genetic similarity with the discovery dataset, potentially replicating false associations.

In another analysis, we constructed a weighted genetic risk score (wGRS) based on *AGXT2* variants for each of the MCI-MRS and for BAIBA: the wGRS was a weighted sum of the effect alleles of the 2 or 7 genome-wide significant variants or 7 variants (for MCI-MRS and BAIBA, respectively), with weights being their estimated effect sizes from the GWAS. These wGRSs were constructed and their associations with MCI were estimated in the HCHS/SOL dataset that was separate from the dataset with metabolomics (set C from Fig. 1). The goal of this analysis was to potentially increase power by aggregating information across SNPs.

### Generalization of SNP associations with MCI in the ARIC study and meta-analysis

We further evaluated the generalization of the significantly associated SNPs in the ARIC longitudinal cohort study (Fig. 1, Step 3) comprising two major US race/ethnic groups, European and African Americans [29, 30]. The protocol for MCI/dementia diagnosis in ARIC has been previously described [31] and is provided in Supplementary Note 1. Data from ARIC visit 5 were, which includes MCI assessment, used in this analysis. Next, we meta-analyzed the results from HCHS/SOL Hispanic/Latino individuals, ARIC European, and ARIC African Americans in an inverse-variance, fixed-effect meta-analysis. To conclude the significance of association while controlling the FWER on the results from the meta-analysis, a *p* value of 0.05/10 = 0.005 is required for a given association.

### Mediation analyses

Mediation analyses were conducted to further examine the relationship between the two variants associated with MCI in replication meta-analysis, and to explore whether these associations are mediated by BAIBA. We used the R “mediation” package, with a complex survey design from the R “survey” package [32], with a “quasibinomial” family for binary traits. This method accounts for the stratification, clustering, and probability weighting in HCHS/SOL to allow correct generalizations to the target population of Latinos in the US. Models were adjusted for age, sex, and study center. A total of *n* = 1490 HCHS/SOL participants with genetic, metabolite, and MCI data were included in the analysis (Fig. 1, Step 4).

### Lifestyle associations with MRS-MCI and BAIBA

We further explored the associations of lifestyle characteristics with MCI-MRS and BAIBA. We used the complex survey design as described above, with the number of participants varying between 3525–3978, depending on the tested lifestyle characteristic, which included: depression, education, physical activity, sleep duration, insomnia, respiratory event index, BMI, smoking, alcohol consumption, and Mediterranean diet score (more information in Supplementary note 2). We computed estimated effect sizes and two-sided Wald test *p* values and noted significance at the nominal *p* value <0.05 level, and computed the required *p* value threshold for controlling the FWER when testing two metabolite measures (MCI-MRS and BAIBA) and ten lifestyle characteristics as 0.05/(2 × 10) = 0.0025.

## Results

Table 1 characterizes the demographic, health, and lifestyle characteristics of the subsets of HCHS/SOL individuals used for the various analyses. Overall, more than 60% of the participants are females, with a weighted mean age of 55 years at visit 1 for the samples of individuals with MCI measures (SOL-INCA participants), and a weighted mean age of 45 years for the subset used for GWAS, including SOL-INCA and younger HCHS/SOL participants. MCI prevalence, measured at the SOL-INCA visit (~7 years after visit 1), is ~10.5%.

**Table 1:** Demographics, health, and lifestyle characteristics of the HCHS/SOL study population datasets.

	Construction of MRS (Sample set A)	GWAS for BAIBA (Sample set B)	GWAS for MRS (Sample set B)	Association of MRS/BAIBA genetic top hits with MCI (Sample set C)	Association of MRS/BAIBA genetic top hits with MCI in unrelated individuals (Sample set D)
*N*	1451	3862	3890	3149	2733
Sex = M (%)	558 (48.1)	1648 (42.7)	1658 (42.6)	1158 (36.8)	1036 (37.9)
Age (years) (mean (SD))	56.03 (8.18)	45.87 (13.83)	45.85 (13.81)	55.23 (7.17)	55.27 (7.18)
Education years (%)					
<12	553 (34.8)	1393 (36.1)	1403 (36.1)	1265 (40.2)	1092 (40.0)
12	324 (21.7)	989 (25.6)	999 (25.7)	686 (21.8)	588 (21.5)
>12	574 (43.4)	1474 (38.2)	1482 (38.2)	1198 (38.0)	1053 (38.5)
Self-reported background (%)					
Dominican	155 (11.3)	378 (9.8)	382 (9.8)	292 (9.3)	243 (8.9)
Central American	150 (7.6)	393 (10.2)	397 (10.2)	302 (9.6)	266 (9.7)
Cuban	266 (26.3)	655 (17.0)	656 (16.9)	609 (19.3)	542 (19.8)
Mexican	518 (30.6)	1385 (35.9)	1399 (36.0)	1094 (34.7)	940 (34.4)
Puerto Rican	251 (16.1)	695 (18.0)	701 (18.0)	537 (17.1)	466 (17.1)
South American	85 (4.3)	225 (5.8)	227 (5.8)	253 (8.0)	226 (8.3)
More than one heritage/Other	25 (3.8)	131 (3.4)	128 (3.3)	62 (2.0)	50 (1.8)
BMI (kg/m^2^) (%)					
Normal weight (<25.0)	244 (16.8)	803 (20.9)	810 (20.9)	489 (15.6)	431 (15.8)
Overweight (25.0–30.0)	568 (39.2)	1405 (36.5)	1417 (36.6)	1281 (40.8)	1114 (40.9)
Obese (>30)	639 (44.0)	1641 (42.6)	1649 (42.5)	1371 (43.6)	1181 (43.3)
Smoking (%)					
Never	823 (55.1)	2261 (58.6)	2279 (58.6)	1775 (56.4)	1523 (55.7)
Former	359 (25.0)	764 (19.8)	772 (19.9)	796 (25.3)	699 (25.6)
Current	269 (19.9)	834 (21.6)	836 (21.5)	578 (18.4)	511 (18.7)
Type II diabetes (%)	376 (27.8)	729 (18.9)	732 (18.8)	886 (28.1)	754 (27.6)
APOE alleles (%)					
23	113 (7.7)	298 (7.7)	300 (7.7)	228 (8.1)	203 (8.3)
24	11 (0.8)	31 (0.8)	31 (0.8)	40 (1.4)	31 (1.3)
33	1008 (69.5)	2666 (69.1)	2691 (69.3)	1934 (69.0)	1665 (68.5)
34	285 (19.6)	788 (20.4)	789 (20.3)	553 (19.7)	489 (20.1)
44	28 (2.0)	60 (1.6)	59 (1.5)	42 (1.5)	40 (1.6)
22	6 (0.4)	15 (0.4)	15 (0.4)	7 (0.2)	4 (0.2)
*APOE*-Ɛ4 carriers (%)	329 (21.8)	879 (22.8)	879 (22.6)	635 (22.6)	560 (23.0)
MCI (%)	163 (11.4)	169 (11.4)	169 (11.4)	328 (10.4)	283 (10.4)

*MRS* metabolomic risk score, *BIABA* beta-aminoisobutyric acid, *MCI* mild cognitive impairment, *SD* standard deviation, *BMI* body mass index.

(%) based on the sampling weights and complex survey design.

All measures are provided from HCHS/SOL visit 1 other than MCI status, inferred at the SOL-INCA exam, on average 7 years after visit 1.

### Heritability estimates for MCI-MRS and BAIBA

The estimated heritability for MCI-MRS was 0.43 (95% CI: 0.19–0.66). The estimated heritability of the BAIBA metabolite was 0.39 (95% CI:0.16–0.61).

### GWAS for MCI-MRS and BAIBA (Fig. 1, step 1)

**Fig. 2:**
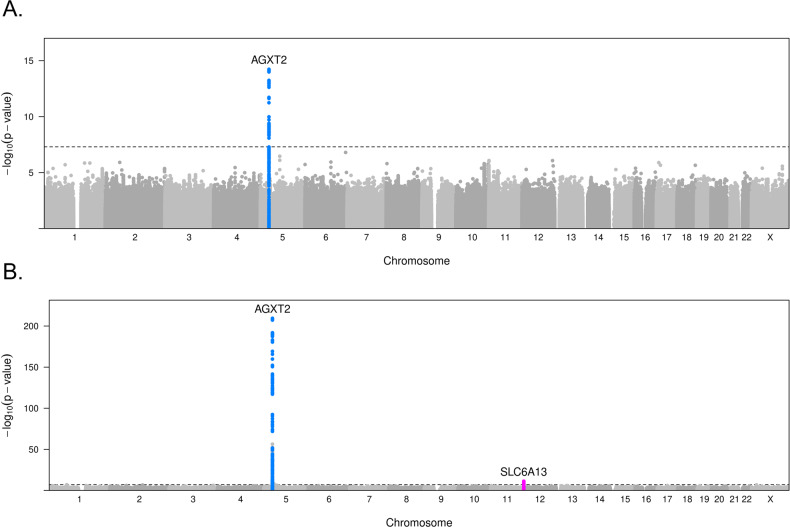
Manhattan plots from GWAS of MCI-MRS and of BAIBA. Manhattan plots of GWAS for **A** MCI-MRS (*n* = 3890) and **B** BAIBA (*n* = 3863) in HCHS/SOL. Every point corresponds to a genetic variant, and the height of the point is the −log(association *p* value) from the MCI-MRS or BAIBA association analysis with *p* values from the score test.

GWAS results for MCI-MRS and BAIBA are presented in Fig. 2 (Manhattan plots) and Supplementary Fig. 1 (QQ-plots). At the significance level of 5 × 10^−8^, 66 variants were significantly associated with MCI-MRS. All significant variants are located in one region, chr5p13.2. The sequential conditional analysis identified two independent variants in this region, with the lead variant, rs37371, having a *p* value = 1.75 × 10^−15^ (Table 2). These two variants explained 2.5% of the residual variance of the MCI-MRS after accounting for baseline covariates, including genetic PCs. This region encompasses the *AGXT2* gene, SNPs in this gene were previously shown to have a strong association with the BAIBA metabolite in plasma and urine [33, 34]. BAIBA is one of the metabolites included in the MCI-MRS and is strongly correlated with the MRS (raw Pearson’s *R* = −0.25, *p* < 2.3e-59).

**Table 2:** Annotation of independent genome-wide significant associations for MCI-MRS and BAIBA in HCHS/SOL sample set B.

							Effect allele frequency in Hispanic/Latino ancestry						
Outcome	Variant	Chromosome	Position (hg38)	Eff. allele	Non-eff. allele	^R2^	European	African	Amerindian	Beta	*p* value	Gene	Annotation
MCI-MRS (*n* = 3890)	rs37371	5	35040125	G	A	0.995	0.920	0.983	0.412	0.232	1.76E-15	*AGXT2*	intron
	rs344515	5	35025665	A	G	0.994	0.621	0.633	0.876	−0.192	1.80E-14	*AGXT2*	intron
BAIBA (*n* = 3863)	rs16899972	5	34998673	A	C	1.000	0.509	0.287	0.475	0.287	4.75E-38	*AGXT2*	missense
	rs183958240	5	35002671	G	A	0.947	0.010	0.010	0.031	−0.811	1.81E-12	*AGXT2*	missense
	rs13174311	5	35032240	T	C	0.998	0.355	0.100	0.023	−0.321	4.13E-32	*AGXT2*	intron
	rs180749	5	35033500	A	G	1.000	0.990	0.816	0.908	1.423	1.63E-192	*AGXT2*	missense
	rs37369	5	35037010	T	C	0.998	0.070	0.639	0.576	0.385	1.93E-51	*AGXT2*	splice donor
	rs140156063	5	35037031	A	G	0.998	0.010	0.012	0.019	1.148	6.00E-14	*AGXT2*	missense
	rs37370	5	35039381	T	C	1.000	0.921	0.984	0.414	−0.892	3.59E-210	*AGXT2*	intron
	rs11613331	12	242301	A	G	0.997	0.515	0.236	0.036	0.168	5.59E-12	*SLC6A13*	intron

Independent associations were identified by sequential conditional analysis.

*MCI* mild cognitive impairment, *MRS* metabolomic risk score, *AF* allele frequency.

*P* values were computed based on the score test.

R^2^ provides the imputation quality of the genetic variant in the HCHS/SOL dataset.

Therefore, we next performed a GWAS for BAIBA. At the significance level of 5 × 10^−8^, 460 variants were significantly associated with BAIBA. Significant variants are located in two loci, chr5p13.2 and chr12p13.33, with the lead variant, rs37370 having *p* value = 3.57 × 10^−210^. Sequential conditional analyses identified eight independent variants, seven of which are located in the *AGXT2* gene (chr5p13.2), and one located in the *SLC6A13* gene in region chr12p13.33 (Table 2). The eight variants explained 34.1% of the residual variance of BAIBA after accounting for covariates, including genetic PCs. Notably, this is only a little lower than the percent variance explained by all additive common genetic effects (heritability, 39%). Three of the seven variants in the *AGXT2* region, that was also associated with the MRS, were in linkage disequilibrium (LD; *R*^2^ = 0.3, 0.6, and 1) with the two SNPs associated with the MRS (Supplementary Table 2). A locus-zoom plot of the top-hit region, chr5p13.2, for BAIBA, is presented in Supplementary Fig. 2. Annotation of the significant variants for both MRS-MCI and BAIBA GWAS is presented in Table 2, together with ancestry-specific frequencies estimations, for the three Latino/Hispanic ancestries (European, African, and Amerindian) [35]. All variants identified in sequential conditional analyses were imputed, with high imputation quality, *R*^2^ > 0.9. Finally, to assess our BAIBA GWAS results in light of other reported GWAS, we looked up a BAIBA GWAS reported in *n* = 6,138 Finnish individuals [36]. This manuscript reported the same association regions detected in our GWAS, and also reported a third, weaker association region, on chr2q22.1. The most significant SNP was rs11127048, which was nominally associated with BAIBA in our data with *p* value = 0.001. Its imputation quality was also high.

### Associations of MCI-MRS-associated SNPs with MCI-MRS metabolites

Supplementary Table 3 reports the associations of the 61 MCI-MRS metabolites with the two SNPs identified at the *AGXT2* region as associated with the MCI-MRS. At the genome-wide significance level, two metabolites were associated with these SNPs: BAIBA, with highly significant associations, and dimethylarginine (both symmetric and nonsymmetric quantified together), with slightly weaker (yet still strong; as measured by *p* values) associations. Notably, like BAIBA, dimethylarginines are substrates of *AGXT2* [37]. Associations of other metabolites with the MCI-MRS SNPs were weak.

### Genetic association analysis with MCI in a separate HCHS/SOL dataset (Fig. 1, step 2)

Comprehensive results from the association analysis of the ten SNPs reported in Table 2 with MCI in an independent subset of HCHS/SOL (*n* = 3149) are presented in Supplementary Table 4. In the baseline model, three of the variants located in the *AGXT2* gene are significantly associated with MCI (*p* < 0.05) in the total subset (rs16899972, rs13174311, and rs140156063). Of these, rs140156063, also passes the FWER threshold (*p* value <0.005). In model 2, which further adjusts for the *APOE* alleles status, all three variants remain significant. After stratification to *APOE*-Ɛ4 carriers and non-carriers, one variant remains significant in the non-carriers (rs140156063), and one variant remains significant in the *APOE*-Ɛ4 carriers (rs16899972). Two other variants, rs37371 and rs37370, were associated with MCI in the *APOE*-Ɛ4 non-carriers subset only. In a secondary analysis, which excludes third-degree related individuals (*n* = 2733, sample set D), effect estimates are similar to those from the primary analysis, and *p* values are slightly changed (as expected).

Supplementary Table 5 reports the association of wGRS for MCI-MRS and for BAIBA with MCI in the HCHS/SOL dataset separate from that used for metabolomics analysis. The associations were highly non-significant with *p* values>0.6.

### Generalization of SNP associations with MCI in the ARIC study and meta-analysis

Supplementary Table 6 characterizes the demographic, health, and lifestyle characteristics of the two ARIC study populations. Overall, almost 70% of the African American and 56% of the European American participants are females. The mean age in both groups is ~79 years, thus this is an older population compared to the SOL-INCA population. As expected, MCI prevalence is higher than that of the relatively younger SOL-INCA population, reaching 20.6% in African Americans and 28.5% in European Americans.

Supplementary Table 7 summarizes the generalization results of the 10 identified SNP associations with MCI, in ARIC European and African American populations, including stratification by *APOE*-Ɛ4 carrier status. Table 3 summarizes the meta-analysis results for all replication datasets, and Supplementary Table 8 provides similar results from analysis stratified by *APOE*-Ɛ4 carrier status. Two of the variants significantly replicate in the ARIC African American subset and remain significant in the meta-analysis; rs16899972 and rs37369. The association between rs16899972 and MCI is driven by the *APOE*-ε4 carrier stratum and the association for rs37369 is driven by the *APOE*-ε4 non-carrier stratum. Two other variants replicate in the European American ARIC subset, only in the *APOE*-Ɛ4 carriers (rs37371 and rs37370), but they do not remain significant in the meta-analysis. Finally, none of the associations reported in the meta-analysis passes the FWER significance threshold requiring *p* value <0.005.

**Table 3:** Replication meta-analysis results for the genetic variants detected as associated with MCI-MRS and BAIBA in HCHS/SOL set B.

	HCHS/SOL		ARIC									
	Set C		African American		European American							
	(*n* = 3149)		(*n* = 1032)		(*n* = 3775)		Meta-analysis					
SNP	Beta	*p* value	Beta	*P* value	Beta	*P* value	Beta	SE	*P* value	Direction	Number of strata	*N* total
rs37371	0.148	1.66E-01	−0.607	7.42E-02	0.060	5.15E-01	0.069	0.068	3.12E-01	+-+	3	7956
rs344515	0.057	5.33E-01	0.041	7.19E-01	0.023	6.69E-01	0.033	0.043	4.42E-01	+++	3	7956
rs16899972	0.184	2.90E-02	0.269	2.01E-02	0.047	3.74E-01	0.110	0.042	8.71E-03	+++	3	7956
rs183958240	0.202	6.50E-01	−12.168	9.74E-01	−11.431	9.60E-01	0.202	0.445	6.50E-01	+--	3	7956
rs13174311	−0.231	2.51E-02	−0.088	6.13E-01	−0.028	6.09E-01	−0.074	0.047	1.14E-01	---	3	7956
rs180749	−0.293	1.27E-01	0.062	6.67E-01	0.149	8.53E-01	−0.062	0.114	5.87E-01	-++	3	7956
rs37369	0.144	1.38E-01	0.254	2.32E-02	0.061	5.23E-01	0.143	0.058	1.40E-02	+++	3	7956
rs140156063	1.722	4.32E-03	−0.027	9.41E-01			0.441	0.312	1.58E-01	+-?	2	4181
rs37370	0.150	1.61E-01	−0.607	7.42E-02	0.060	5.15E-01	0.070	0.068	3.08E-01	+-+	3	7956
rs11613331	−0.047	6.06E-01	0.110	3.63E-01	0.097	6.82E-02	0.067	0.043	1.21E-01	-++	3	7956

Inverse-variance fixed-effects meta-analysis was used to meta-analyze results from the three replication datasets: HCHS/SOL set C, and ARIC African and European Americans.

Effect sizes and SEs were estimated based on the mixed-models analysis approach.

*P* values from tests of variant associations in each stratum were based on the score test.

Heterogeneity of effects between datasets was low (*p* value >0.05 based on the Cochran’s heterogeneity test) for all variants.

### Mediation analyses

**Fig. 3:**
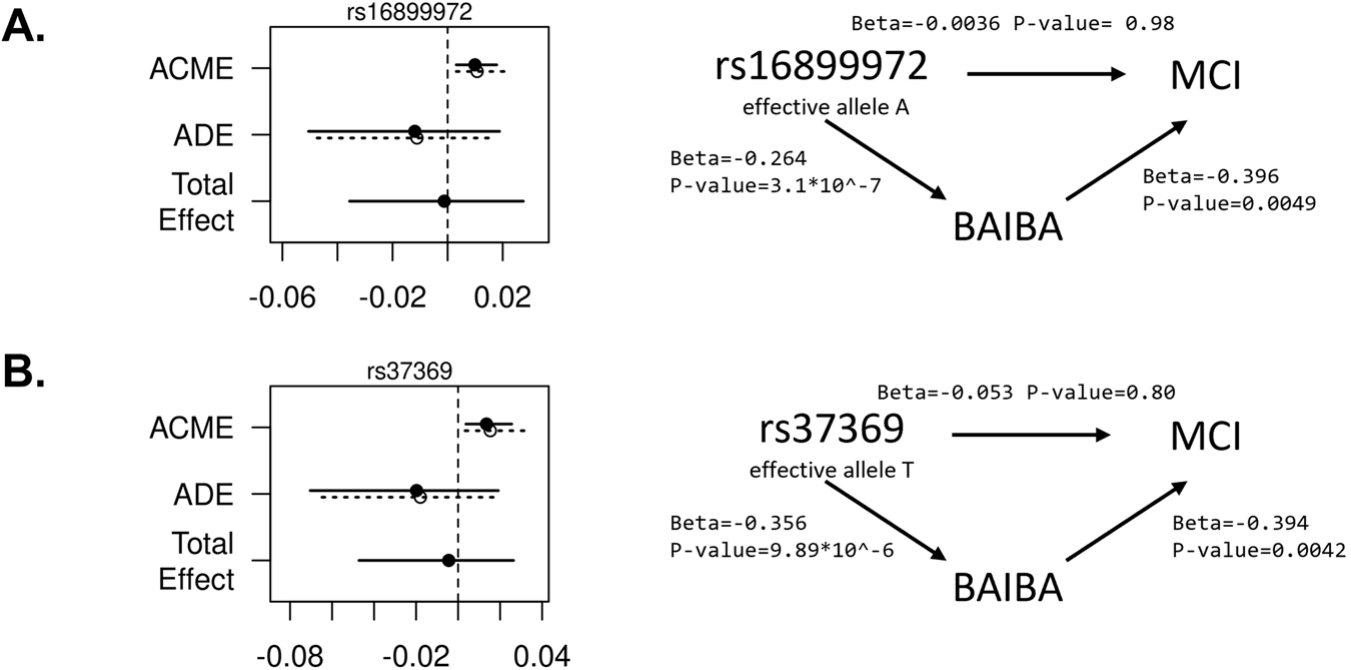
Visualization of results from mediation analysis using BAIBA. Mediation analysis results for two genetic variants associated with MCI, mediated by BAIBA **A** rs16899972, **B** rs37369. Note that the main effect is not significant as expected by the earlier report of the association between the variant and MCI in set D (*n* = 3178), since the mediation analysis was conducted in a smaller sample, similar to Set A (*n* = 1490). Effect estimates and *p* values were obtained from the R median package. ACME average causal mediation effects and ADE average direct effects.

Mediation results are illustrated in Fig. 3, suggesting that BAIBA is a causal mediator between the two genetic variants; rs16899972 and rs37369, and MCI. Average causal mediation effects (ACME) are significant for both variants (*p* value = 0.004), whereas average direct effects (ADE) are non-significant. We could not quantify the proportion of mediated effect based on these results because the estimated ADE has an opposite direction of association to the estimated ACME.

### Lifestyle associations with MRS-MCI and BAIBA

Association results of lifestyle characteristics are summarized in Supplementary Table 9. Statistically significant results at the *p* < 0.05 level include depression, associated with higher MCI-MRS, education which is associated with lower MCI-MRS, and both higher respiratory event index and BMI are associated with reduced BAIBA levels. However, none of the associations passed the FWER control *p* value threshold of 0.0025.

## Discussion

We studied the genetic determinants of a previously developed MCI-MRS in Hispanic/Latino older adults in the United States and their association with MCI in this population and other US populations. GWAS for the MCI-MRS highlighted a locus located on chr5p13.2, encompassing the *AGXT2* gene, previously known as associated with BAIBA. BAIBA is a highly heritable metabolite included in the MCI-MRS, inversely associated with MCI risk. Further investigation of the genetic components of this metabolite confirmed a previously known highly significant association of the chr5p13.2 locus with BAIBA and an additional association region on chr12p13.33, encompassing the *SLC6A13* gene. We identified via sequential conditional analysis 2 independent SNPs associated with MCI-MRS and 8 independent variants associated with BAIBA. Meta-analysis of the association of these variants with MCI in an independent subset of SOL-INCA, and ARIC European and African Americans, highlighted two variants located in the *AGXT2* gene: rs16899972, a missense variant, and rs37369, a splice donor variant. Mediation analysis suggested that these genetic variants contribute via changes in BAIBA levels to MCI development.

BAIBA is a non-protein amino acid secreted by skeletal muscles upon regular exercise, causing the browning of white adipose tissue and an increase in thermogenesis, thus benefiting other tissues and organs in an endocrine manner [38]. It was shown that plasma BAIBA concentrations are increased with exercise and are inversely associated with cardiometabolic risk factors such as fasting glucose, insulin sensitivity, triglycerides, total cholesterol, BMI, and inflammatory reactions [33]. Our results align with the known high heritability of BAIBA, with a striking association between the variants in the *AGXT2* loci and BAIBA plasma levels, accounting for a substantial portion of the heritability [39]. The *AGXT2* gene encodes the enzyme alanine-glyoxylate aminotransferase 2, which catalyzes the transamination between BAIBA and pyruvate [33]. Several studies have found an association between rs37369, one of the significant variants in our meta-analysis, associated with BAIBA levels in White individuals [40]. This polymorphism constitutes a nonsynonymous valine-to-isoleucine (V140I) substitution in the *AGXT2* protein [34]. The association of rs37369 was weak in ARIC European American compared to African American and HCHS/SOL Hispanic/Latino individuals, perhaps due to reduced power due to allele frequencies differences: 0.1, 0.36, and 0.55 in European, Hispanic/Latino, and African Americans, respectively. Mediation analysis suggests that the association between this variant (and the other identified variant) and MCI is mediated by BAIBA. The result in our previously published paper supports the hypothesis that BAIBA has a protective effect against MCI (both for the direct effect of BAIBA in the MCI-MRS and individually).

The top SNP from the second region associated with BAIBA, chr12p13.33, encompassing the *SLC6A13* gene, was not associated with MCI, as expected, since this loci was not associated with MRS-MCI. This variant was previously associated with the level of BAIBA and other metabolites in European and Hispanic populations [41, 42]. We estimated the associations of the two SNPs associated with MCI from the *AGXT2* region with all other metabolites composing the MCI-MRS. These two SNPs were also associated with dimethylarginines, which are substrates of *AGXT2*. The metabolite measuring the two dimethylarginines (symmetric and asymmetric) was not associated with MCI in our previous analysis [14] when accounting for multiple testing, though it did have a nominal association where higher levels of dimethylarginines were associated with reduced MCI risk with *p* value = 0.03 (see summary statistics here: https://github.com/chloehe1129/Metabolomics-on-CogDec/blob/main/MCI/MCI_associations_all_participants_mdl1.csv). We did not further pursue the analysis of dimethylarginines despite previous literature linking these chemicals to dementia and AD; previous studies are focused on asymmetric dimethylarginine [43, 44], and the reported associations are sometimes of the opposite direction of what we see in SOL-INCA—higher asymmetric dimethylarginine levels in plasma increase risk of cognitive decline [45, 46]. In all, perhaps we have a lower power to detect associations with MCI because both the asymmetric and symmetric dimethylarginines are measured together.

Our study has a few limitations. First, the detected associations with MCI do not pass multiple testing adjustment. While we are confident in the results due to the careful process of hypothesis generation starting from metabolite-MCI association analysis, identification of strong associations of metabolite measures with genetic loci, followed by analysis in three independent datasets, which resulted in consistent findings, future studies should further validate the two associations that had *p* value <0.05 in the validation meta-analysis, and perhaps further study this association region. Second, similar to other epidemiological studies, the metabolite identification pipeline used by Metabolon does not distinguish between the two compounds D-BAIBA and L-BAIBA, which are involved in different metabolism and downstream effects [24]. Similarly, it does not distinguish between symmetric and asymmetric dimethylarginine, which may have a role in cognitive aging as well. Their effects on MCI may differ, future studies are needed to assess their specific effects and to explore causal inference for BAIBA on MCI in larger sample sizes. Third, the mediation analysis used the same dataset that identified the association of both the MCI-MRS and of BAIBA with MCI. This may lead to over-estimation of the average causal mediated effect.

Overall, we identified a genomic association region for MCI-MRS, with two variants associated with MCI in Hispanic/Latino, European, and African Americans. These variant associations support BAIBA as a metabolite with a protective effect on MCI development 7 years after metabolite assessment.

## Supplementary information

Supplementary Materials

## Data Availability

HCHS/SOL genetic, phenotypic, and metabolomics data can be obtained through the study’s Data Coordinating Center using an approved data use agreement. Information is provided at https://sites.cscc.unc.edu/hchs/. HCHS/SOL genetic and phenotypic data can also be obtained from dbGaP under accession number phs000810.v1.p1. ARIC genetic and phenotypic data can be obtained through the study’s Coordinating Center using an approved data use agreement. Information is provided at https://sites.cscc.unc.edu/aric/distribution-agreements. ARIC genetic and phenotypic data can also be obtained from dbGaP under accession number phs000280.v1.p1.

## References

[1] Alzheimer’s Association. 2020 Alzheimer’s disease facts and figures. Alzheimers Dement. 2020;16:391–460.

[2] Winblad B, Palmer K, Kivipelto M, Jelic V, Fratiglioni L, Wahlund LO (2004). Mild cognitive impairment—beyond controversies, towards a consensus: report of the International Working Group on Mild Cognitive Impairment. J Intern Med.

[3] Mufson EJ, Binder L, Counts SE, DeKosky ST, de Toledo-Morrell L, Ginsberg SD (2012). Mild cognitive impairment: pathology and mechanisms. Acta Neuropathol.

[4] Logue MW, Panizzon MS, Elman JA, Gillespie NA, Hatton SN, Gustavson DE (2019). Use of an Alzheimer’s disease polygenic risk score to identify mild cognitive impairment in adults in their 50s. Mol Psychiatry.

[5] Chaudhury S, Brookes KJ, Patel T, Fallows A, Guetta-Baranes T, Turton JC (2019). Alzheimer’s disease polygenic risk score as a predictor of conversion from mild-cognitive impairment. Transl Psychiatry.

[6] Rodríguez-Rodríguez E, Sánchez-Juan P, Vázquez-Higuera JL, Mateo I, Pozueta A, Berciano J (2013). Genetic risk score predicting accelerated progression from mild cognitive impairment to Alzheimer’s disease. J Neural Transm.

[7] González HM, Tarraf W, Schneiderman N, Fornage M, Vásquez PM, Zeng D (2019). Prevalence and correlates of mild cognitive impairment among diverse Hispanics/Latinos: Study of Latinos-Investigation of Neurocognitive Aging results. Alzheimers Dement.

[8] Granot-Hershkovitz E, Tarraf W, Kurniansyah N, Daviglus M, Isasi CR, Kaplan R (2020). APOE alleles’ association with neurocognitive function differ across Hispanic background groups. Alzheimer’s Dement.

[9] Sofer T, Kurniansyah N, Granot-Hershkovitz E, Goodman MO, Tarraf W, Broce I, et al. Polygenic risk scores for Alzheimer’s disease and mild cognitive impairment in Hispanics/Latinos in the U.S: the study of Latinos—Investigation of Neurocognitive Aging. medRxiv. 2021:2021.01.08.21249413.

[10] Trivedi DK, Hollywood KA, Goodacre R (2017). Metabolomics for the masses: the future of metabolomics in a personalized world. N. Horiz Transl Med.

[11] Yan X, Hu Y, Wang B, Wang S, Zhang X (2020). Metabolic dysregulation contributes to the progression of Alzheimer’s disease. Front Neurosci.

[12] Jiang Y, Zhu Z, Shi J, An Y, Zhang K, Wang Y (2019). Metabolomics in the development and progression of dementia: a systematic review. Front Neurosci.

[13] Tynkkynen J, Chouraki V, van der Lee SJ, Hernesniemi J, Yang Q, Li S (2018). Association of branched-chain amino acids and other circulating metabolites with risk of incident dementia and Alzheimer’s disease: a prospective study in eight cohorts. Alzheimers Dement.

[14] He S, Granot-Hershkovitz E, Zhang Y, Bressler J, Tarraf W, Yu B (2022). Blood metabolites predicting mild cognitive impairment in the study of Latinos-investigation of neurocognitive aging (HCHS/SOL). Alzheimers Dement.

[15] Zheng J, Haberland V, Baird D, Walker V, Haycock PC, Hurle MR (2020). Phenome-wide Mendelian randomization mapping the influence of the plasma proteome on complex diseases. Nat Genet.

[16] Porcu E, Gilardi F, Darrous L, Yengo L, Bararpour N, Gasser M (2021). Triangulating evidence from longitudinal and Mendelian randomization studies of metabolomic biomarkers for type 2 diabetes. Sci Rep..

[17] Lavange LM, Kalsbeek WD, Sorlie PD, Aviles-Santa LM, Kaplan RC, Barnhart J (2010). Sample design and cohort selection in the Hispanic Community Health Study/Study of Latinos. Ann Epidemiol.

[18] Sorlie PD, Aviles-Santa LM, Wassertheil-Smoller S, Kaplan RC, Daviglus ML, Giachello AL (2010). Design and implementation of the Hispanic Community Health Study/Study of Latinos. Ann Epidemiol.

[19] González HM, Tarraf W, Fornage M, González KA, Chai A, Youngblood M (2019). A research framework for cognitive aging and Alzheimer’s disease among diverse US Latinos: Design and implementation of the Hispanic Community Health Study/Study of Latinos-Investigation of Neurocognitive Aging (SOL-INCA). Alzheimers Dement.

[20] Albert MS, DeKosky ST, Dickson D, Dubois B, Feldman HH, Fox NC (2011). The diagnosis of mild cognitive impairment due to Alzheimer’s disease: recommendations from the National Institute on Aging-Alzheimer’s Association workgroups on diagnostic guidelines for Alzheimer’s disease. Alzheimers Dement.

[21] Tomaszewski Farias S, Mungas D, Harvey DJ, Simmons A, Reed BR, Decarli C (2011). The measurement of everyday cognition: development and validation of a short form of the Everyday Cognition scales. Alzheimers Dement.

[22] González HM, Tarraf W, Gouskova N, Gallo LC, Penedo FJ, Davis SM (2015). Neurocognitive function among middle-aged and older Hispanic/Latinos: results from the Hispanic Community Health Study/Study of Latinos. Arch Clin Neuropsychol.

[23] Tibshirani R (1996). Regression shrinkage and selection via the lasso. J R Stat Soc Ser B.

[24] González HM, Tarraf W, Jian X, Vásquez PM, Kaplan R, Thyagarajan B (2018). Apolipoprotein E genotypes among diverse middle-aged and older Latinos: study of Latinos-Investigation of Neurocognitive Aging results (HCHS/SOL). Sci Rep..

[25] Kowalski MH, Qian H, Hou Z, Rosen JD, Tapia AL, Shan Y (2019). Use of >100,000 NHLBI trans-omics for precision medicine (TOPMed) Consortium whole genome sequences improves imputation quality and detection of rare variant associations in admixed African and Hispanic/Latino populations. PLoS Genet.

[26] Conomos MP, Reiner AP, Weir BS, Thornton TA (2016). Model-free estimation of recent genetic relatedness. Am J Hum Genet.

[27] Conomos MP, Laurie CA, Stilp AM, Gogarten SM, McHugh CP, Nelson SC (2016). Genetic diversity and association studies in US Hispanic/Latino populations: applications in the Hispanic Community Health Study/Study of Latinos. Am J Hum Genet.

[28] Sofer T, Zheng X, Gogarten SM, Laurie CA, Grinde K, Shaffer JR, et al. A fully adjusted two-stage procedure for rank-normalization in genetic association studies. Genet Epidemiol. 2019;43:263–75. 10.1002/gepi.22188 PMC6416071 30653739

[29] Rebholz CM, Yu B, Zheng Z, Chang P, Tin A, Köttgen A (2018). Serum metabolomic profile of incident diabetes. Diabetologia..

[30] Bressler J, Yu B, Mosley TH, Knopman DS, Gottesman RF, Alonso A (2017). Metabolomics and cognition in African American adults in midlife: the atherosclerosis risk in communities study. Transl Psychiatry.

[31] Knopman DS, Gottesman RF, Sharrett AR, Wruck LM, Windham BG, Coker L (2016). Mild cognitive impairment and dementia prevalence: the atherosclerosis risk in communities neurocognitive study (ARIC-NCS). Alzheimers Dement.

[32] Lumley T, Scott A (2017). Fitting regression models to survey data. Stat Sci.

[33] Tanianskii DA, Jarzebska N, Birkenfeld AL, O’Sullivan JF, Rodionov RN. Beta-aminoisobutyric acid as a novel regulator of carbohydrate and lipid metabolism. Nutrients. 2019;11:524. 10.3390/nu11030524 PMC6470580 30823446

[34] Suhre K, Wallaschofski H, Raffler J, Friedrich N, Haring R, Michael K (2011). A genome-wide association study of metabolic traits in human urine. Nat Genet.

[35] Granot-Hershkovitz E, Sun Q, Argos M, Zhou H, Lin X, Browning SR (2022). AFA: ancestry-specific allele frequency estimation in admixed populations: The Hispanic Community Health Study/Study of Latinos. HGG Adv.

[36] Yin X, Chan LS, Bose D, Jackson AU, VandeHaar P, Locke AE (2022). Genome-wide association studies of metabolites in Finnish men identify disease-relevant loci.. Nat Commun..

[37] Kittel A, Müller F, König J, Mieth M, Sticht H, Zolk O (2014). Alanine-glyoxylate aminotransferase 2 (AGXT2) polymorphisms have considerable impact on methylarginine and β-aminoisobutyrate metabolism in healthy volunteers. PLoS ONE.

[38] Roberts LD, Boström P, O’Sullivan JF, Schinzel RT, Lewis GD, Dejam A (2014). β-Aminoisobutyric acid induces browning of white fat and hepatic β-oxidation and is inversely correlated with cardiometabolic risk factors. Cell Metab.

[39] Rhee EP, Ho JE, Chen MH, Shen D, Cheng S, Larson MG (2013). A genome-wide association study of the human metabolome in a community-based cohort. Cell Metab.

[40] Rodionov RN, Jarzebska N, Weiss N, Lentz SR (2014). AGXT2: a promiscuous aminotransferase. Trends Pharm Sci.

[41] Shin SY, Fauman EB, Petersen AK, Krumsiek J, Santos R, Huang J (2014). An atlas of genetic influences on human blood metabolites. Nat Genet.

[42] Feofanova EV, Chen H, Dai Y, Jia P, Grove ML, Morrison AC (2020). A genome-wide association study discovers 46 loci of the human metabolome in the Hispanic Community Health Study/Study of Latinos. Am J Hum Genet.

[43] Asif M, Soiza RL, McEvoy M, Mangoni AA (2013). Asymmetric dimethylarginine: a possible link between vascular disease and dementia. Curr Alzheimer Res.

[44] Abe T, Tohgi H, Murata T, Isobe C, Sato C (2001). Reduction in asymmetrical dimethylarginine, an endogenous nitric oxide synthase inhibitor, in the cerebrospinal fluid during aging and in patients with Alzheimer’s disease. Neurosci Lett.

[45] Arlt S, Schulze F, Eichenlaub M, Maas R, Lehmbeck JT, Schwedhelm E (2008). Asymmetrical dimethylarginine is increased in plasma and decreased in cerebrospinal fluid of patients with Alzheimer’s disease. Dement Geriatr Cogn Disord.

[46] Selley ML (2003). Increased concentrations of homocysteine and asymmetric dimethylarginine and decreased concentrations of nitric oxide in the plasma of patients with Alzheimer’s disease. Neurobiol Aging.

