# Supplementary material for: Genetic loci of beta-aminoisobutyric acid are associated with aging-related mild cognitive impairment
Source: Transl Psychiatry. 2023 Apr 29;13:140. doi: 10.1038/s41398-023-02437-y (PMC10148805; doi:10.1038/s41398-023-02437-y)
Supplement: Supplementary file 1 — Supplementary Materials [file 41398_2023_2437_MOESM1_ESM.pdf]

Genetic loci of beta-aminoisobutyric acid are associated with aging-related Mild Cognitive Impairment: Supplementary Materials

Einat Granot-Hershkovitz, Brian Spitzer, Yunju Yang, Wassim Tarraf, Bing Yu, Eric Boerwinkle, Myriam Fornage, Thomas H. Mosley, Charles DeCarli, Bruce Kristal, Hector M Gonzalez, Tamar Sofer

Supplementary Note 1: ARIC Methods 2

Supplementary Note 2: Lifestyle characteristics included in the association analysis with MRS-MCI and BAIBA 3

Supplementary Tables 4

Supplementary Table 1: MRS metabolites and weights 4

Supplementary Table 2: LD between *AGXT2* region MRS SNPs and BAIBA SNPs 6

Supplementary Table 3: Association of MCI-MRS associated SNPs with MCI-MRS metabolites7

Supplementary Table 4: Association of the significant GWAS loci identified in HCHS/SOL set B with MCI in HCHS/SOL replication samples. 9

Supplementary Table 5: Estimated associations of wGRS with MCI in HCHS/SOL set B 10

Supplementary Table 6: Demographics, health, and lifestyle characteristics of the ARIC study population datasets 11

Supplementary Table 7: Associations of the variants that were associated with MCI-MRS and BAIBA in HCHS/SOL set B with MCI in ARIC, stratified by APOE-Ɛ4 carrier status. 12

Supplementary Table 8: Replication meta-analysis results for the genetic variants detected as associated with MCI-MRS and BAIBA in HCHS/SOL set B, stratified by *APOE*-Ɛ4 carrier status. 13

Supplementary Table 9: Association between lifestyle characteristics and MRS for MCI or BAIBA in HCHS/SOL (n=3978) 14

Supplementary Figures 15

Supplementary Figure 1: QQ-plots of GWAS for MCI-MRS and BAIBA in HCHS/SOL (n=3,862). 15

Supplementary Figure 2: Locus zoom plot for the top locus, chr5p13.2, associated with BAIBA in n=3,862 HCHS/SOL participants. 16

Supplementary References 17

Supplementary Note 1: ARIC Methods

The Atherosclerosis Risk in Communities (ARIC) study is a prospective longitudinal study of the development of atherosclerosis, and its clinical sequelae in which 15,792 individuals aged 45-64 years from four communities in the United States were enrolled at the baseline examination (1987-1989). A detailed description of the ARIC study has been reported prevmiously[1]. Various medical information and specimens were collected at each of the eight examinations. Written informed consent was provided by all study participants, and the study design and methods were approved by the institutional review boards at each of the collaborating medical institutions: University of Mississippi Medical Center Institutional Review Board (Jackson Field Center); Wake Forest University Health Sciences Institutional Review Board (Forsyth County Field Center); University of Minnesota Institutional Review Board (Minnesota Field Center); and Johns Hopkins University School of Public Health Institutional Review Board (Washington County Field Center). In this analysis, we use 3,775 European American and 1,032 African American ARIC participants with Mild Cognitive Impairment (MCI) status determined based on data from ARIC visit 5. Detailed descriptions of participants' health, demographic, and lifestyle characteristics are presented below.

The protocol for MCI and dementia diagnosis in ARIC has been previously described in detail[2]. At visit 5, all participants were identified as cognitively normal or possible MCI/dementia cases with the use of an algorithm that considered MMSE scores, Clinical Dementia Rating sum of boxes, concurrent performance on the neuropsychological test battery, and change in cognitive function from previous assessments. All cases that were identified as possible MCI/dementia by this algorithm and a sample of cognitively normal participants were reviewed by 2 experts (a physician and a neuropsychologist), who classified participants' cognitive status as normal, MCI, or dementia, with discordant diagnoses adjudicated by a third reviewer.

Supplementary Note 2: Lifestyle characteristics included in the association analysis with MRS-MCI and BAIBA

Depression (10-item Center for Epidemiologic Studies Depression Scale (CES-D) summary score), education (three levels:<12 yrs, 12 yrs, <12yrs), physical activity (Total physical activity (min/day)), sleep duration (Average sleep duration (hours)), insomnia (8 Women's Health Initiative Insomnia Rating Scale), respiratory event index (Apnea/Hypopnea Index (3% desat)), BMI (kg/m^2), smoking (three levels: Never, Former, Current), alcohol consumption (three levels: Never, Former, Current), Mediterranean diet score. The Mediterranean dietary score is derived from the mean intakes of food groups/nutrients from two 24-hour dietary recalls, ranging between 0 (lowest adherence to the diet) and 9 (highest adherence to the diet) [3].

Supplementary Tables

Supplementary Table 1: MRS metabolites and weights

Biochemical name	HMDB ID	Weight	% missing
Adenosine	HMDB00050	-0.067	4.32%
Alpha-ketobutyrate	HMDB00005	0.132	21.09%
2-aminoadipate	HMDB00510	0.076	0.53%
3-aminoisobutyrate	HMDB03911	-0.112	0.23%
2-aminooctanoate	HMDB00991	-0.025	10.46%
Betaine	HMDB00043	-0.029	0.05%
Carotene diol (1)		0.043	0.20%
Carotene diol (2)		0.040	16.09%
Cerotoylcarnitine (C26)*	HMDB06347	0.064	11.97%
Cysteine-glutathione disulfide	HMDB00656	-0.024	0.55%
12,13-DiHOME	HMDB04705	-0.131	16.57%
1-dihomo-linolenylglycerol (20:3)		0.039	2.46%
2,3-dihydroxy-2-methylbutyrate	HMDB29576	0.030	7.79%
1,2-dilinoleoyl-GPC (18:2/18:2)	HMDB08138	-0.081	0.08%
Dimethylarginine (SDMA + ADMA)	HMDB01539	-0.024	0.10%
Dimethylglycine	HMDB00092	-0.087	0.10%
Docosadioate		-0.017	1.76%
Eicosanodioate		-0.129	0.10%
Gamma-glutamylhistidine		-0.123	0.10%
Glutamate, gamma-methyl ester	HMDB61715	-0.051	0.80%
Glutarylcarnitine (C5-DC)	HMDB13130	-0.100	0.13%
Glycochenodeoxycholate sulfate		0.223	4.78%
Glycodeoxycholate	HMDB00631	0.048	21.04%
Glycosyl ceramide (d18:1/20:0, d16:1/22:0)*		-0.044	5.15%
Glycosyl-N-(2-hydroxynervonoyl)-sphingosine (d18:1/24:1(2OH))*		0.009	19.13%
Guanidinoacetate	HMDB00128	0.148	0.15%
Hexadecanedioate	HMDB00672	0.064	0.25%
2-hydroxyoctanoate	HMDB02264	-0.024	2.49%
3-hydroxypyridine sulfate		0.024	2.01%
Hyocholate	HMDB00760	-0.050	11.84%
Isobutyrylglycine	HMDB00730	0.094	15.96%
Lactosyl-N-palmitoyl-sphingosine (d18:1/16:0)		-0.059	0.10%

Linoleoyl-linoleoyl-glycerol (18:2/18:2) [1]*	HMDB07248	-0.058	1.99%
Maleate	HMDB00176	0.036	1.48%
Methionine	HMDB00696	-0.023	0.00%
N('1)-acetylspermidine	HMDB01276	-0.010	0.43%
N1-methylinosine	HMDB02721	-0.011	0.83%
N6,N6,N6-trimethyllysine	HMDB01325	-0.064	0.10%
N6-succinyladenosine	HMDB00912	0.075	12.92%
N-acetylglucosaminylasparagine	HMDB00489	-0.062	11.54%
N-trimethyl 5-aminovalerate		0.070	0.10%
Oleoyl ethanolamide	HMDB02088	0.020	0.60%
1-oleoyl-GPE (18:1)	HMDB11506	0.061	0.18%
2'-O-methyluridine		0.130	3.47%
Orotate	HMDB00226	-0.035	0.18%
5-oxoproline	HMDB00267	-0.014	0.00%
1-palmitoleoyl-GPC (16:1)*	HMDB10383	0.035	0.10%
Phenylacetylcarnitine		0.050	2.84%
Phenylalanylglycine	HMDB28995	-0.017	21.27%
Picolinate	HMDB02243	0.039	5.88%
Pimeloylcarnitine/3-methyladipoylcarnitine (C7-DC)		-0.026	13.10%
Pyroglutamine*		-0.001	0.10%
Quinolinate	HMDB00232	-0.189	0.10%
Sphingomyelin (d18:1/25:0, d19:0/24:1, d20:1/23:0, d19:1/24:0)*		-0.117	1.99%
Stachydrine	HMDB04827	0.033	0.10%
1-stearoyl-2-oleoyl-GPS (18:0/18:1)	HMDB10163	0.146	24.26%
Suberate (octanedioate)	HMDB00893	0.082	15.74%
Taurolithocholate 3-sulfate	HMDB02580	0.003	4.93%
Trigonelline (N'-methylnicotinate)	HMDB00875	0.033	0.10%
Tryptophan betaine	HMDB61115	-0.141	0.35%
Ursodeoxycholate	HMDB00946	-0.141	11.97%

Abbreviations: *MRS* Metabolite risk score, *HMDB* Human Metabolome Database.
*Metabolite compounds that have not been officially confirmed based on a standard, but Metabolon Inc. is confident in their identities.

Supplementary Table 2: LD between *AGXT2* region MRS SNPs and BAIBA SNPs

SNP	Chr	Position	Effect allele	Non effect allele	R^2
		LD with MRS SNP rs37371			
rs16899972	5	34998673	A	C	0.00
rs183958240	5	35002671	G	A	0.00
rs13174311	5	35032240	T	C	0.05
rs180749	5	35033500	A	G	0.01
rs37369	5	35037010	T	C	0.30
rs140156063	5	35037031	A	G	0.00
rs37370	5	35039381	T	C	1.00
		LD with MRS SNP rs344515			
rs16899972	5	34998673	A	C	0.07
rs183958240	5	35002671	G	A	0.02
rs13174311	5	35032240	T	C	0.64
rs180749	5	35033500	A	G	0.13
rs37369	5	35037010	T	C	0.02
rs140156063	5	35037031	A	G	0.01
rs37370	5	35039381	T	C	0.08

rs37371 is on chromosome 5, position 35040125, effect allele G, other (non-effect) allele is A.
rs344515 is on chromosome 5, position 35025665, effect allele A, other allele (non-effect) is G.
Coordinates are provided in GRCh38i assembly.

Supplementary Table 3: Association of MCI-MRS associated SNPs with MCI-MRS metabolites

		rs344515		rs37371	
**Metabolite**	**sample size**	**Effect est**	**p-value**	**Effect est**	**p-value**
Adenosine	3697	-0.032	0.192	0.052	0.066
Alpha-ketobutyrate	3043	0.010	0.730	-0.020	0.530
2-aminoadipate	3842	0.013	0.566	-0.004	0.882
3-aminoisobutyrate	3854	0.595	1.25E-131**	-0.899	1.10E-211**
2-aminooctanoate	3461	0.011	0.662	0.065	0.024*
Betaine	3860	0.000	0.990	-0.003	0.917
Carotene diol (1)	3855	0.005	0.815	0.024	0.365
Carotene diol (2)	3242	-0.002	0.927	-0.009	0.768
Cerotoylcarnitine (C26)*	3409	-0.039	0.116	-0.005	0.872
Cysteine-glutathione disulfide	3842	-0.016	0.506	0.017	0.537
12,13-DiHOME	3225	0.008	0.777	0.000	0.995
1-dihomo-linolenylglycerol (20:3)	3766	-0.049	0.046	0.004	0.896
2,3-dihydroxy-2-methylbutyrate	3557	-0.018	0.451	0.011	0.692
1,2-dilinoleoyl-GPC (18:2/18:2)	3859	-0.035	0.135	0.001	0.957
Dimethylarginine (SDMA + ADMA)	3859	0.124	3.75E-08**	-0.265	1.06E-23**
Dimethylglycine	3859	0.017	0.477	-0.025	0.373
Docosadioate	3795	0.030	0.218	0.015	0.601
Eicosanodioate	3859	0.011	0.621	0.015	0.562
Gamma-glutamylhistidine	3859	-0.007	0.751	0.007	0.787
Glutamate, gamma-methyl ester	3832	-0.006	0.797	-0.009	0.734
Glutarylcarnitine (C5-DC)	3858	0.009	0.710	-0.022	0.404
Glycochenodeoxycholate sulfate	3680	0.054	0.031*	-0.002	0.952
Glycodeoxycholate	3050	0.043	0.111	-0.009	0.768
Glycosyl ceramide (d18:1/20:0, d16:1/22:0)*	3670	-0.002	0.928	-0.021	0.465
Glycosyl-N-(2-hydroxynervonoyl)-sphingosine (d18:1/24:1(2OH))*	3134	-0.058	0.021*	0.029	0.315
Guanidinoacetate	3857	-0.022	0.292	0.047	0.055
Hexadecanedioate	3853	-0.037	0.130	0.016	0.564
2-hydroxyoctanoate	3766	0.024	0.324	0.000	0.989
3-hydroxypyridine sulfate	3787	-0.003	0.879	-0.043	0.108
Hyocholate	3405	0.032	0.223	0.028	0.347
Isobutyrylglycine	3249	0.027	0.327	-0.040	0.201
Lactosyl-N-palmitoyl-sphingosine (d18:1/16:0)	3859	-0.017	0.480	0.024	0.403
Linoleoyl-linoleoyl-glycerol (18:2/18:2) [1]*	3789	-0.044	0.074	0.023	0.432
Maleate	3804	0.003	0.894	0.029	0.297
Methionine	3862	-0.005	0.810	-0.036	0.169

N('1)-acetylspermidine	3846	0.033	0.178	-0.026	0.351
N1-methylinosine	3832	-0.009	0.683	-0.013	0.633
N6,N6,N6-trimethyllysine	3859	0.057	0.014*	0.008	0.765
N6-succinyladenosine	3358	0.010	0.697	0.028	0.327
N-acetylglucosaminylasparagine	3419	0.017	0.476	-0.015	0.592
N-trimethyl 5-aminovalerate	3859	-0.026	0.267	-0.043	0.109
Oleoyl ethanolamide	3839	-0.024	0.324	0.003	0.904
1-oleoyl-GPE (18:1)	3856	-0.031	0.192	0.041	0.131
2'-O-methyluridine	3732	0.006	0.807	0.004	0.893
Orotate	3856	0.008	0.734	0.029	0.316
5-oxoproline	3862	-0.009	0.717	0.012	0.674
1-palmitoleoyl-GPC (16:1)*	3859	-0.044	0.064	0.015	0.586
Phenylacetylcarnitine	3755	-0.030	0.226	-0.046	0.110
Phenylalanylglycine	3038	0.017	0.553	0.042	0.184
Picolinate	3636	0.030	0.216	-0.012	0.675
Pimeloylcarnitine/3-methyladipoylcarnitine (C7-DC)	3354	0.025	0.330	0.025	0.409
Pyroglutamine*	3859	0.034	0.113	-0.022	0.388
Quinolinate	3859	0.079	0.001*	-0.058	0.043*
Sphingomyelin (d18:1/25:0, d19:0/24:1, d20:1/23:0, d19:1/24:0)*	3786	-0.025	0.314	0.006	0.821
Stachydrine	3858	0.001	0.978	0.014	0.608
1-stearoyl-2-oleoyl-GPS (18:0/18:1)	2934	-0.042	0.134	0.004	0.902
Suberate (octanedioate)	3251	-0.026	0.302	-0.005	0.866
Taurolithocholate 3-sulfate	3670	0.016	0.516	-0.001	0.973
Trigonelline (N'-methylnicotinate)	3859	-0.009	0.684	0.019	0.464
Tryptophan betaine	3850	0.027	0.265	-0.005	0.854
Ursodeoxycholate	3401	-0.016	0.535	0.009	0.775

We estimated the association of each of the two MCI-MRS *AGXT2* SNPs with each of the metabolites composing the MCI-MRS in linear mixed models using complete (not imputed) metabolites. Models were adjusted for age, sex, study center, 5 PCs of genetic data, and genetic analysis group, and random effects accounted for genetic relatedness, household sharing, and block unit sharing. P-values are from the 1-degrees of freedom score test. Analyses were performed using the GENESIS R package.

Supplementary Table 4: Association of the significant GWAS loci identified in HCHS/SOL set B with MCI in HCHS/SOL replication samples.

						Sample set C								Sample set D	
						Model 1						Model 2		Model 1	
						Total (n = 3,149)		*APOE-Ɛ4* non-carriers (n = 2,169)		*APOE-Ɛ4* carriers (n = 635)		Total (n = 2,804)		Total (n = 2,733)	
Previous GWAS Outcome	Variant	Chrosome	Position (hg38)	Eff. allele	Non eff. allele	Beta	p-value	Beta	p-value	Beta	p-value	Beta	p-value	Beta	p-value
MRS	rs37371	5	35040125	G	A	0.148	1.66E-01	0.281	3.60E-02	-0.148	5.49E-01	0.170	1.43E-01	0.162	1.56E-01
	rs344515	5	35025665	A	G	0.057	5.33E-01	0.030	7.92E-01	0.237	2.59E-01	0.071	4.70E-01	0.052	5.95E-01
BAIBA	rs16899972	5	34998673	A	C	0.184	2.90E-02	0.108	2.96E-01	0.472	1.26E-02	0.197	2.81E-02	0.204	2.32E-02
	rs183958240	5	35002671	G	A	0.202	6.50E-01	0.173	7.70E-01	0.338	7.94E-01	0.238	6.58E-01	0.412	3.84E-01
	rs13174311	5	35032240	T	C	-0.231	2.51E-02	-0.246	5.11E-02	-0.357	1.21E-01	-0.264	1.56E-02	-0.210	5.77E-02
	rs180749	5	35033500	A	G	-0.293	1.27E-01	-0.405	8.90E-02	-0.264	5.61E-01	-0.378	7.02E-02	-0.271	1.84E-01
	rs37369	5	35037010	T	C	0.144	1.38E-01	0.090	4.55E-01	0.169	4.43E-01	0.121	2.46E-01	0.101	3.32E-01
	rs140156063	5	35037031	A	G	1.722	4.32E-03	2.020	5.71E-03	1.411	2.25E-01	1.897	2.19E-03	1.487	2.55E-02
	rs37370	5	35039381	T	C	0.150	1.61E-01	0.282	3.45E-02	-0.146	5.54E-01	0.172	1.37E-01	0.164	1.50E-01
	rs11613331	12	242301	A	G	-0.047	6.06E-01	-0.083	4.51E-01	-0.003	9.87E-01	-0.053	5.80E-01	-0.115	2.41E-01

Abbreviations: *MCI* : mild cognitive impairment; *MRS* : metabolic risk score.
Model 1: Adjusted for age, sex, center, genetic analysis groups, first 5 PCs of genetic data.
Model 2: Model 1 with further adjustment for *APOE* -ε4 and *APOE* -ε2 carrier status.

Supplementary Table 5: Estimated associations of wGRS with MCI in HCHS/SOL set B

wGRS	Effect estimate	SE	P-value
MCI-MRS	0.59	1.19	0.62
BAIBA	0.08	0.90	0.93

The effect sizes are the log-odds ratios estimated in logistic mixed models of a wGRS in association with MCI in HCHS/SOL set B (the set of individuals with genetic data, MCI status, and no metabolomics). SE is the standard error estimate of the effect estimate, and p-value is the corresponding p-value.

The MCI-MRS wGRS was constructed based on two *AGXT2* SNPs independently associated with the MCI-MRS in conditional analysis.

The BABIBA wGRS was constructed based on seven *AGXT2* SNPs independently associated with the BAIBA in repeated conditional analyses

Supplementary Table 6: Demographics, health, and lifestyle characteristics of the ARIC study population datasets

	African American	European
**N**	1,032	3,775
**SEX = M (%)**	323 (31.30%)	1653 (43.79%)
**AGE (years) (mean (SD))**	78.57 (5.15)	79.38(5.17)
**Education years (%)**		
**<12**	286(27.71%)	308 (8.16%)
**12**	217 (21.03%)	1319 (34.94%)
**>12**	529 (51.26%)	2148 (56.90%)
**BMI (kg/m2) (%)**		
**Normal weight (< 25.0)**	162 (15.70%)	1015 (26.89%)
**Overweight (25.0 – 30.0)**	334 (32.36%)	1431 (37.91%)
**Obese (> 30)**	468 (45.35%)	1104 (29.25%)
**Smoking (%)**		
**Never**	376 (36.43%)	1310 (34.70%)
**Former**	391 (37.89%)	1831 (48.50%)
**Current**	66 (6.40%)	208 (5.51%)
***APOE*-Ɛ4 carriers (%)**	378 (35.8%)	939 (23.9%)
**MCI (%)**	213 (20.6%)	268 (28.5%)

Abbreviations: *ARIC*: Atherosclerosis Risk In Communities; *SD*: standard deviation: *BMI*: body mass index; *MCI*: mild cognitive impairment.

Supplementary Table 7: Associations of the variants that were associated with MCI-MRS and BAIBA in HCHS/SOL set B with MCI in ARIC, stratified by APOE-Ɛ4 carrier status.

						Effective allele frequency in ARIC		African American						European American					
								Total (n=1,032)		*APOE-Ɛ4* non Carriers (n=654)		*APOE- Ɛ4* Carriers (n=378)		Total (n=3,775)		*APOE-Ɛ4* non Carriers (n=2,836)		*APOE-Ɛ4* Carriers (n=939)	
Previous GWAS Outcome	Variant	Chrosome	Position (hg38)	Eff. allele	Non eff. allele	African Americans	European American	Beta	P-value	Beta	P-value	Beta	P-value	Beta	P-value	Beta	P-value	Beta	P-value
MRS	rs37371	5	35040125	G	A	0.980	0.909	-0.607	7.416E-02	-0.330	4.994E-01	-0.880	8.130E-02	0.060	5.154E-01	-0.063	5.618E-01	0.477	1.674E-02
	rs344515	5	35025665	A	G	0.638	0.599	0.041	7.193E-01	0.124	4.200E-01	-0.034	8.417E-01	0.023	6.689E-01	0.039	5.412E-01	0.046	6.537E-01
BAIBA	rs16899972	5	34998673	A	C	0.330	0.518	0.269	2.009E-02	0.164	2.791E-01	0.451	1.610E-02	0.047	3.745E-01	0.007	9.131E-01	0.168	1.035E-01
	rs183958240	5	35002671	G	A	0.001	0.000	-12.168	9.744E-01	-12.061	9.820E-01	-13.286	9.880E-01	-11.431	9.603E-01	-11.255	9.724E-01	-12.677	9.811E-01
	rs13174311	5	35032240	T	C	0.116	0.342	-0.088	6.134E-01	-0.149	5.127E-01	-0.029	9.171E-01	-0.028	6.089E-01	-0.034	6.124E-01	-0.083	4.401E-01
	rs180749	5	35033500	A	G	0.817	0.999	0.062	6.669E-01	0.246	2.264E-01	-0.117	5.805E-01	0.149	8.528E-01	0.449	6.824E-01	-0.209	8.656E-01
	rs37369	5	35037010	T	C	0.551	0.079	0.254	2.316E-02	0.253	7.976E-02	0.258	1.553E-01	0.061	5.228E-01	0.192	8.472E-02	-0.298	1.367E-01
	rs140156063	5	35037031	A	G	0.024	0.000	-0.027	9.412E-01	0.020	9.667E-01	-0.109	8.534E-01						
	rs37370	5	35039381	T	C	0.980	0.909	-0.607	7.416E-02	-0.330	4.994E-01	-0.880	8.130E-02	0.060	5.154E-01	-0.063	5.618E-01	0.477	1.674E-02
	rs11613331	12	242301	A	G	0.278	0.543	0.110	3.628E-01	0.121	4.422E-01	0.086	6.649E-01	0.097	6.821E-02	0.090	1.561E-01	0.161	1.294E-01

Supplementary Table 8: Replication meta-analysis results for the genetic variants detected as associated with MCI-MRS and BAIBA in HCHS/SOL set B, stratified by *APOE*-Ɛ4 carrier status.

	Total datasets									*APOE* -Ɛ4 non Carriers									*APOE* -Ɛ4 Carriers								
SNP	Beta	SE	Z	P-value Z	direction	Q	pval.Q	Number of strata	N total	Beta	SE	Z	P-value Z	direction	Q	pval.Q	Number of strata	N total	Beta	SE	Z	P-value Z	direction	Q	pval.Q	Number of strata	N total
rs37371	0.069	0.068	1.01	3.12E-01	+-+	4.510	1.05E-01	3	7,956	0.062	0.083	0.74	4.59E-01	+--	4.641	9.82E-02	3	5,659	0.135	0.148	0.91	3.64E-01	--+	8.301	1.58E-02	3	1,952
rs344515	0.033	0.043	0.77	4.42E-01	+++	0.110	9.47E-01	3	7,956	0.047	0.053	0.90	3.69E-01	+++	0.287	8.66E-01	3	5,659	0.057	0.082	0.70	4.85E-01	+-+	1.027	5.99E-01	3	1,952
rs16899972	0.110	0.042	2.62	8.71E-03	+++	4.079	1.30E-01	3	7,956	0.050	0.051	0.97	3.31E-01	+++	1.332	5.14E-01	3	5,659	0.278	0.081	3.41	6.49E-04	+++	3.043	2.18E-01	3	1,952
rs183958240	0.202	0.445	0.45	6.50E-01	+--	0.004	9.98E-01	3	7,956	0.173	0.592	0.29	7.70E-01	+--	0.002	9.99E-01	3	5,659	0.338	1.294	0.26	7.94E-01	+--	0.001	1.00E+00	3	1,952
rs13174311	-0.074	0.047	-1.58	1.14E-01	---	3.030	2.20E-01	3	7,956	-0.085	0.057	-1.48	1.38E-01	---	2.288	3.19E-01	3	5,659	-0.121	0.092	-1.31	1.89E-01	---	1.285	5.26E-01	3	1,952
rs180749	-0.062	0.114	-0.54	5.87E-01	-++	2.253	3.24E-01	3	7,956	-0.019	0.153	-0.12	9.01E-01	-++	4.511	1.05E-01	3	5,659	-0.144	0.189	-0.76	4.45E-01	---	0.089	9.56E-01	3	1,952
rs37369	0.143	0.058	2.46	1.40E-02	+++	1.722	4.23E-01	3	7,956	0.171	0.071	2.41	1.61E-02	+++	0.810	6.67E-01	3	5,659	0.051	0.115	0.45	6.54E-01	++-	4.622	9.92E-02	3	1,952
rs140156063	0.441	0.312	1.41	1.58E-01	+-?	6.153	1.31E-02	2	4,181	0.629	0.403	1.56	1.19E-01	++?	5.210	2.25E-02	2	2,823	0.201	0.525	0.38	7.02E-01	+-?	1.360	2.43E-01	2	1,013
rs37370	0.070	0.068	1.02	3.08E-01	+-+	4.536	1.04E-01	3	7,956	0.063	0.083	0.76	4.49E-01	+--	4.691	9.58E-02	3	5,659	0.135	0.148	0.91	3.62E-01	--+	8.285	1.59E-02	3	1,952
rs11613331	0.067	0.043	1.55	1.21E-01	-++	2.010	3.66E-01	3	7,956	0.055	0.052	1.06	2.90E-01	-++	2.051	3.59E-01	3	5,659	0.114	0.083	1.37	1.71E-01	-++	0.620	7.33E-01	3	1,952

Inverse-variance fixed-effects meta-analysis was used to meta-analyzed results from the three replication datasets: HCHS/SOL set C, and ARIC African and European Americans.
Effect sizes and SEs were estimated based on the mixed-models analysis approach.

Supplementary Table 9: Association between lifestyle characteristics and MRS for MCI or BAIBA in HCHS/SOL (n=3978)

Exposure	Outcome	N	Beta	SE	P-value
Depression (10-item CES-D summary score)	MRS - MCI	3,923	0.00016	0.00007	2.99E-02
Education (Three levels:<12 yrs, 12 yrs, <12yrs)		3,962	-0.00102	0.00049	3.82E-02
Physical activity (Total physical activity (min/day))		3,954	0.00000	0.00000	1.07E-01
Sleep Duration (Average sleep duration (hours))		3,768	-0.00006	0.00032	8.47E-01
Insomnia (8 Womens's Health Initiative Insomnia Rating Scale)		3,863	0.00009	0.00007	2.34E-01
Respratory event index (Apnea/Hypopnea Index (3% desat))		3,525	0.00006	0.00004	1.54E-01
BMI (kg/m^2)		3,954	-0.00007	0.00009	4.54E-01
Smoking (Three levels: Never, Former,Current)		3,965	-0.00037	0.00065	5.71E-01
Drinking (Three levels: Never, Former,Current)		3,967	-0.00080	0.00068	2.37E-01
Mediterranean diet score		3,968	-0.00014	0.00031	6.53E-01
Depression (10-item CES-D summary score)	BAIBA	3,923	-0.00357	0.00324	2.71E-01
Education (Three levels:<12 yrs, 12 yrs, <12yrs)		3,962	-0.01137	0.02446	6.42E-01
Physical activity (Total physical activity (min/day))		3,954	0.00001	0.00002	6.11E-01
Sleep Duration (Average sleep duration (hours))		3,768	0.02191	0.01556	1.60E-01
Insomnia (8 Womens's Health Initiative Insomnia Rating Scale)		3,863	-0.00044	0.00372	9.05E-01
Respratory event index (Apnea/Hypopnea Index (3% desat))		3,525	-0.00475	0.00174	6.65E-03
BMI (kg/m^2)		3,954	-0.00892	0.00334	7.72E-03
Smoking (Three levels: Never, Former,Current)		3,965	0.03884	0.02542	1.27E-01
Drinking (Three levels: Never, Former,Current)		3,967	0.02759	0.02880	3.38E-01
Mediterranean diet score		3,978	-0.00502	0.01491	7.36E-01

Supplementary Figures

Supplementary Figure 1: QQ-plots of GWAS for MCI-MRS and BAIBA in HCHS/SOL (n=3,862).

(a) QQ-plot of the MCI-MRS GWAS

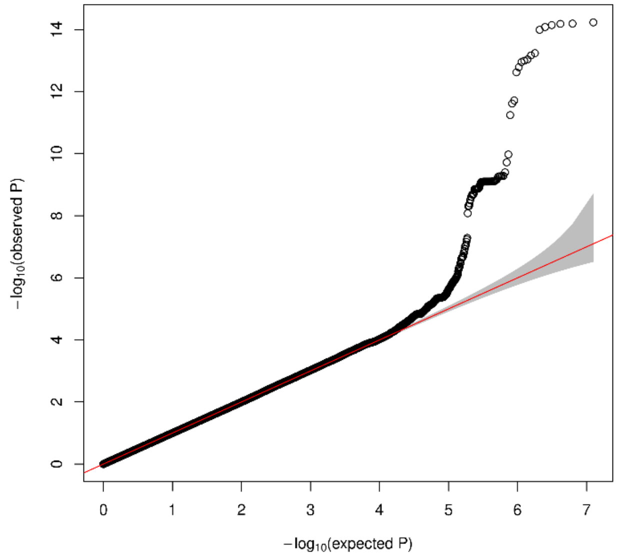

(b) QQ-plot of the BAIBA GWAS

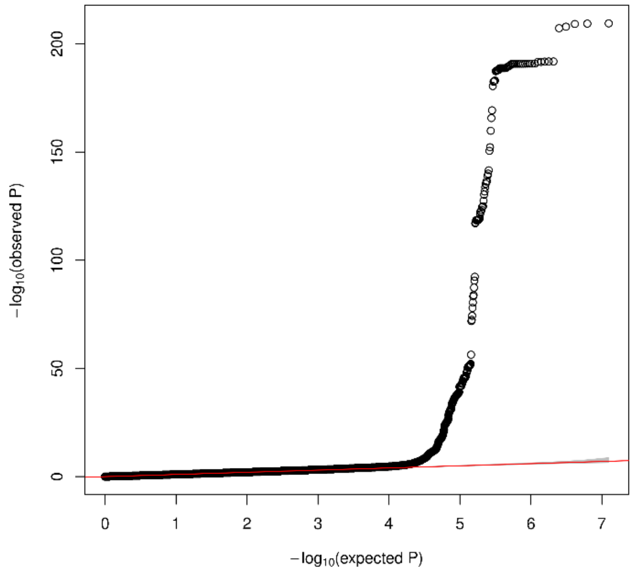

Supplementary Figure 2: Locus zoom plot for the top locus, chr5p13.2, associated with BAIBA in n=3,862 HCHS/SOL participants.

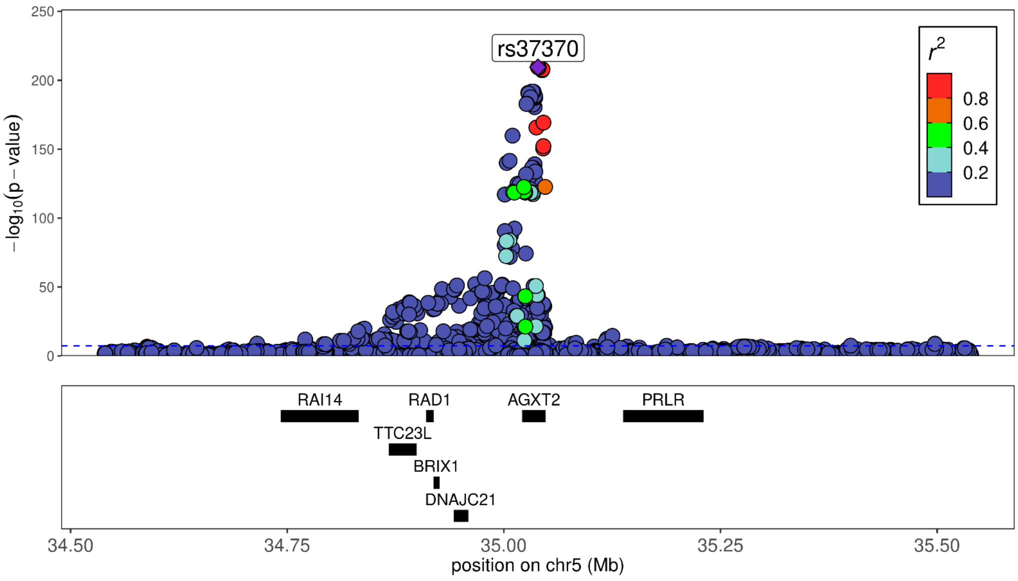

The purple diamond is the most statistically significant SNP in the region, rs37370. All other SNPs are shown as circles, in colors that represent the degree of linkage disequilibrium with the index SNP (R2 values).

Supplementary References

1. Wright, J.D., Folsom, A.R., Coresh, J., Sharrett, A.R., Couper, D., Wagenknecht, L.E., Mosley, T.H., Ballantyne, C.M., Boerwinkle, E.A., Rosamond, W.D., et al. (2021). The ARIC (Atherosclerosis Risk In Communities) Study: JACC Focus Seminar 3/8. J. Am. Coll. Cardiol. *77*, 2939–2959.
2. Knopman, D.S., Gottesman, R.F., Sharrett, A.R., Wruck, L.M., Windham, B.G., Coker, L., Schneider, A.L.C., Hengrui, S., Alonso, A., Coresh, J., et al. (2016). Mild cognitive impairment and dementia prevalence: The Atherosclerosis Risk in Communities Neurocognitive Study. Alzheimer's Dement. Diagnosis, Assess. Dis. Monit. *2*, 1–11.
3. Fung, T., Hu, F., Mccullough, M., Newby, P.K., Willett, W.C., and Holmes, M.D. (2006). Diet Quality Is Associated with the Risk of Estrogen Receptor-Negative Breast Cancer in Postmenopausal Women 1. Nutr. Epidemiol. *136*, 466–472.
